# *Practical Approach to Care Kit*: Innovation for nurses’ clinical practice in HIV management

**DOI:** 10.1590/1518-8345.5998.3720

**Published:** 2023-01-30

**Authors:** Ianka Cristina Celuppi, Betina Hörner Schlindwein Meirelles, Veridiana Tavares Costa, Denise Elvira Pires de Pires

**Affiliations:** 1 Universidade Federal de Santa Catarina, Florianópolis, SC, Brazil.; 2 Universidade Federal de Santa Catarina, Laboratório Bridge - CTC, Florianópolis, SC, Brazil.; 3 Scholarship holder at the Coordenação de Aperfeiçoamento de Pessoal de Nível Superior (CAPES), Brazil.; 4 Universidade Federal de Santa Catarina, Departamento de Enfermagem, Florianópolis, SC, Brazil.; 5 Diretoria de Atenção Primária a Saúde, Secretaria de Saúde do Estado de Santa Catarina (DAPS/SES), Florianópolis, SC, Brazil.; 6 Scholarship holder at the Conselho Nacional de Desenvolvimento Científico e Tecnológico (CNPq), Brazil.

**Keywords:** Technology, Practice Guideline, Evidence-Based Practice, HIV, Nursing Care, Disease Management, Tecnologia, Guia de Prática Clínica, Prática Clínica Baseada em Evidências, HIV, Cuidados de Enfermagem, Gerenciamento Clínico, Tecnología, Guía de Práctica Clínica, Práctica Clínica Basada en la Evidencia, VIH, Atención de Enfermería, Manejo de la Enfermedad

## Abstract

**Objective:**

to analyze the use of the *Practical Approach to Care Kit* as a technology adopted in nurses’ clinical practice for HIV management in Primary Health Care.

**Method:**

an exploratory and descriptive research study anchored in the methodological framework of the Constructivist Grounded Theory. The participants were defined through initial sampling, with 12 nurses, and theoretical sampling, with five managers, totaling 17 participants. The data were collected by means of intensive interviews and documentary analysis, and they were analyzed in two stages: 1) Initial coding; and 2) Focused coding.

**Results:**

the professionals identified the *Practical Approach to Care Kit* as a technological innovation that contributed to expanding the clinical practice and to empowering nurses in the clinical management of HIV infection. They also highlighted its importance as a tool for guiding the different responsibilities and duties while sharing care, contributing to the provision of evidence-based practices.

**Conclusion:**

The *Practical Approach to Care Kit* is a technological innovation that has transformed nurses’ clinical practice in HIV management, expanding their scope of activities in carrying out the diagnosis, assessing the health condition and counseling, evaluating adherence to the treatment, adverse effects and prescription of exams, medications, and immunobiological.

## Resumo

**Objetivo:**

analisar a utilização do *Practical Approach to Care Kit* como uma tecnologia adotada na prática clínica dos enfermeiros no manejo do HIV na Atenção Primária à Saúde.

**Método:**

pesquisa exploratória e descritiva, ancorada no referencial metodológico da Teoria Fundamentada nos Dados Construtivista. A definição dos participantes foi realizada por amostragem inicial, com 12 enfermeiros, e amostragem teórica, com cinco gestores, totalizando 17 participantes. Os dados foram coletados por entrevistas intensivas e análise documental, e foram analisados em duas etapas: 1) codificação inicial; e 2) codificação focalizada.

**Resultados:**

os profissionais identificaram o *Practical Approach to Care Kit* como uma inovação tecnológica que contribuiu para a ampliação da prática clínica e empoderamento do enfermeiro no manejo clínico da infecção por HIV. Também destacaram sua importância como ferramenta para orientação das diferentes responsabilidades e atribuições no compartilhamento do cuidado, contribuindo para a prestação de práticas baseadas em evidências.

**Conclusão:**

o *Practical Approach to Care Kit* é uma inovação tecnológica que transformou a prática clínica do enfermeiro no manejo do HIV, ampliando seu escopo de atividades na realização do diagnóstico, avaliação da condição de saúde e aconselhamento, avaliação da adesão ao tratamento, efeitos adversos e prescrição de exames, medicamentos e imunobiológicos.

## Resumen

**Objetivo:**

analizar el uso del *Practical Approach to Care Kit* como tecnología adoptada en la práctica clínica de los enfermeros en el manejo del VIH en la Atención Primaria de la Salud.

**Método:**

investigación exploratoria y descriptiva, basada en el marco metodológico de la Teoría Fundamentada en los Datos Constructivistas. La definición de los participantes fue realizada por muestreo inicial, con 12 enfermeros, y muestreo teórico, con cinco gestores, fueron 17 participantes en total. Los datos fueron recolectados a través de entrevistas intensivas y análisis de documentos, y fueron analizados en dos etapas: 1) codificación inicial; y 2) codificación enfocada.

**Resultados:**

los profesionales identificaron el *Practical Approach to Care Kit* como una innovación tecnológica que contribuyó a la expansión de la práctica clínica y al empoderamiento de los enfermeros en el manejo clínico de la infección por VIH. También destacaron su importancia como herramienta para orientar las diferentes responsabilidades y obligaciones en la distribución de la atención de los pacientes, contribuyendo a la prestación de prácticas basadas en evidencia.

**Conclusión:**

el *Practical Approach to Care Kit* es una innovación tecnológica que ha transformado la práctica clínica de los enfermeros en el manejo del VIH, ampliando su radio de acción para la realización del diagnóstico, evaluación del estado de salud y asesoramiento, evaluación de la adherencia al tratamiento, efectos adversos y prescripción de exámenes, medicamentos e inmunobiológicos.

**Highlights**

(1) The PACK expanded nurses’ clinical performance and autonomy in the management of HIV infection.

(2) With this, nurses perform their clinical practice based on scientific evidence.

(3) Using the PACK has contributed to changing the HIV care model in Florianópolis.

(4) Clinical management of HIV in PHC performed by nurses in Florianópolis is innovative.

## Introduction

Nurses’ clinical practice has exerted a positive impact on health care in the scope of Primary Health Care (PHC) and encompasses care and clinic management actions, involving a complex set of activities supported by evidence-based practices and the use of management microtechnologies^1^ ^-^ ^3^.

A new care model for people living with HIV has been discussed and implemented gradually in the health services This model highlights PHC and turns care into a center for reorienting the HIV management practices, encompassing structuring elements that should be part of the care practices, such as: risk stratification model; qualification of professionals; technical support guarantee; providing access to CD4 and viral load (VL) tests; and enabling access to antiretrovirals (ARVs)^4^.

Although the HIV epidemic in Brazil is considered stable, the infection became a chronic condition that requires the adoption of technologies that strengthen the care practices and strategies for this population segment^4^ ^-^ ^5^. These technologies can be of the material type, such as instruments, tools and devices, or the non-material type, including new ways to organize work, processes, and flows^6^. The technologies present in the health workspace can materialize in the production of relationships between subjects and in knowledge construction, and express themselves in changes in the organizational structures of the health services^7^ ^-^ ^8^.

The *Practical Approach to Care Kit* (PACK) was developed at the University of Cape Town in South Africa and consists of a tool based on recent scientific evidence and global health care recommendations. Its structure is based on four pillars: 1) Guide of clinical guidelines; 2) Professional training strategy; 3) Monitoring; and 4) Implementation of changes for the health system^9^ ^-^ ^10^. The Adult Brazil PACK consists of a technology used in nurses’ clinical practice in PHC, a concise tool to support clinical decision-making, which resorts to algorithms based on symptoms and a standardized checklist to assist physicians and nurses in the evaluation, counseling, and treatment of health conditions^4^ ^,^ ^9^.

The municipality of Florianópolis, Santa Catarina/Brazil, was the first to implement PACK in the country. Using this technology has contributed to the improvement of teamwork, sharing of care, expansion of the nurses’ clinic, and professional training for the management of the main diseases monitored in PHC, such as HIV^10^ ^-^ ^11^. Other international contexts also contribute to successful experiences in using the PACK^12^ ^-^ ^13^.

In view of the above, and considering that the guidelines for the decentralization of HIV care are recent and that, up to date, the clinical management guidelines prepared by the Brazilian Ministry of Health are directed to medical professionals^4^, the need is identified to carry out studies that deal with nurses’ role in the management of people living with HIV in PHC. Consequently, the objective of this study is to analyze the use of the *Practical Approach to Care Kit* as a technology adopted in nurses’ clinical practice for HIV management in Primary Health Care.

## Method

### Study type and design

This is an exploratory and descriptive research study of a qualitative nature, anchored in the methodological framework of the Constructivist Grounded Theory (GT), which seeks to analyze experiences and interactions in order to understand meanings attributed to a phenomenon^14^.

The constructivist strand has symbolic interactionism and social constructivism as its philosophical bases, which presuppose the elaboration of a theory through contact between researcher and participants, allowing data co-creation. Thus, multiple realities and social interactions are acknowledged as important to build the interpretations, in order to urge the researchers’ reflective ability in relation to their analyses^14^.

This manuscript followed the guidelines set forth in the Consolidated criteria for reporting qualitative research (COREQ)^15^.

### Data collection locus

The study was conducted in the municipality of Florianópolis, which presents the highest Human Development Index (HDI-M) among the capital cities of the country (0.847). In 2020, the health-related expenses were R$ 811.73 *per* inhabitant/year. In July 2021, Florianópolis had a total of 150 Family Health Teams, reaching 77.30% of population coverage^16^. The municipal network has 49 Health Centers (HCs) distributed across 4 health districts^17^.

Based on its own criteria, the Florianópolis Municipal Health Department (*Secretaria Municipal de Saúde*, SMS) defined four PHC HCs, one in each health district, to comprise the study setting. After identifying the need to interview new actors, the research was also conducted in five SMS sectors, which were chosen by the researchers during the concomitant data collection and analysis.

### Period

Data collection took place from July to September 2020.

### Selection criteria

The definition of the study participants was first carried out by means of initial sampling and, subsequently, through theoretical sampling, which guides the search for places, actors, and events that enhance the findings and formation of the analysis categories, filling the gaps that arise throughout the research^14^. In the initial sampling, the criteria for selecting the participants and collecting/analyzing preliminary data are established, whereas in the theoretical sampling, conceptual and theoretical refinement of the data is sought.

The groups of participants were intentionally selected, considering their performance in the PHC services and use of the PACK, as well as their contribution to the process of implementing the technological innovation.

The following were defined as inclusion criteria for the initial sampling group: 1) working as a clinical nurse, coordinator, or resident in PHC; and 2) having at least six months of experience in PHC in relation to the data collection date. For the second group theoretical sampling, the inclusion criterion defined was having worked in the Florianópolis SMS management area for more than six months in relation to the data collection date. For both sampling groups, the exclusion criterion considered corresponded to the professionals who were distanced from work during the data collection period, regardless of the reason.

After applying the inclusion and exclusion criteria, the participants were randomly invited to participate in the research, with no prioritization and/or sorting criteria in this stage.

### Participants

In the initial sampling group, 12 nurses who worked in four PHC health centers were interviewed, representing 75% of all the active nurses in these services (n=16). In the theoretical sampling group, five managers who worked in the specialized care, clinical management, care integration, and epidemiological surveillance SMS sectors were interviewed.

### Instruments used for data collection

The data were collected by means of intensive interviews. The following statement was used to start the conversation with the initial sampling group: Talk to me about the care practices targeted at people living with HIV. From data collection with this group, the following hypothesis was proposed: the care practices for people living with HIV were related to the decentralization of clinical management, supported by care protocols and scientific evidence guides.

Thus, the objective of the interviews with the theoretical sampling group consisted in exploring aspects related to the elaboration/institution of clinical management protocols aimed at HIV, such as the PACK, conducted by the initial question: Talk to me about the use of protocols and clinical management guides for best care practices targeted at people living with HIV.

A documentary analysis of the PACK itself was also carried out, in order to investigate the introductory texts of the instrument, the use guidelines and the nurses’ clinical management guidelines for the care of people with HIV and other conditions to be monitored in PHC. The documentary analysis was carried out during the entire data collection and analysis process, assisting in understanding the practices developed by the nurses and in interpreting the participants’ statements.

### Data collection

Data collection was in charge of a single researcher, an auditing nurse, during the MSc in Nursing course at the Federal University of Santa Catarina. The researcher underwent training sessions on the theme of the care to be provided to people living with HIV and on theoretical-methodological guidelines of the Constructivist GT during her participation in the research group’s activities and in graduate courses.

The researcher’s assumptions were around the fact that nurses provided care to people living with HIV with better autonomy and clinical performance level in PHC from Florianópolis. This practice took place in the context of decentralization of HIV clinical management to the PHC services, structuring of the network of services in the municipality, and use of technologies to support clinical decision-making.

To collect data from the interviews of the first group, a semi-structured script was developed aiming at understanding the context in which the care practices were developed, the functions performed by nurses and the tools/technologies they used. For data collection with the second group, an adaptation was made to the interview script aiming at analyzing the new hypotheses, which dealt with the institution of protocols, tools and technologies in health work in PHC. Both versions of the script were validated in the research group and tested with two interviewees (Nurse 01 and Manager 01), with no need for changes/adaptations.

Contact with the participants was made available by SMS, inviting them to participate in the research via messages sent by email or *WhatsApp* ^®^. The intensive interviews took place in a remote modality due to the COVID-19 pandemic, by video calls through the *Google Meet* ^®^ communication system and during the research participants’ working hours. Each interview lasted a mean of 29 minutes.

Data saturation was reached at the end of the interviews with the second sampling group, totaling 17 participants. In qualitative research, data saturation can be understood as the moment in which the categories cease to generate new properties and data collection does not contribute any more elucidations to the thematic under study^14^.

During the interviews, the participants were alone in their rooms, and a few of them were interrupted by co-workers to deal with emerging demands, not causing harm to data collection and analysis. Some nurses showed fatigue due to the work overload resulting from the pandemic, which may have compromised some of their answers to the research script questions. However, it was noticed that the participants liked to participate in the interviews, as they evidenced in their speeches the importance of the research topic and of disseminating the knowledge about the practices developed in Florianópolis.

The interviews were transcribed by the researcher using the *Word* ^®^ text tool and were sent for validation by the participants, who felt satisfied with the description of the meanings attributed to their experiences. Spelling and grammatical adjustments were requested by one participant.

### Data analysis

For the analysis, the data were introduced and organized into the Atlas.ti software, version 9.0, following the guidelines set forth in the Constructivist GT. Data analysis followed two coding phases: 1) Initial coding; and 2) Focused coding. In the first phase, the incidents were coded in order to understand the diverse information based on the participants’ meanings and experiences, constituting the first conceptual dimensions of the analyzed experience. In the second phase, the most expressive codes were grouped to give rise to 28 abstract categories and synthesize the data fragments^14^. Memos and diagrams were also prepared to aid analytical data development. For Charmaz, the constant data comparison process in the GT strengthens the assertions about the implicit data and contributes to reducing the risk of bias in data analysis^14^.

Documentary analysis of the PACK was an important strategy to identify the characteristics of the tool and establish relationships with the data collected in intensive interviews, reflecting nurses’ daily work. The introduction of this second body of knowledge took place at the first interview (E01), in which the PACK was mentioned as a technology used by nurses in the management of HIV infection.

The characterization of the *Practical Approach to Care Kit* as a technological innovation for nurses’ clinical performance in HIV management contributed to understanding the phenomenon: “Unveiling the best management practices in the care of people living with HIV related to decentralized, shared and evidence-based care”.

### Ethical aspects

The research followed the ethical principles recommended at the international level and by the Brazilian norms, as well as it was ethically approved by the UFSC Research Ethics Committee (*Comitê de Ética em Pesquisas*, CEP). The participants’ anonymity was ensured by using codes during data analysis and categorization, where the nurses were assigned Code N (N01, N02, N03) and the managers were represented by Code M (M01, M02, M03). The free and informed consent form was electronically sent to the participants and authorized via *Google Forms* ^®^, with a copy kept by the researcher.

## Results

### Characterization of the participants

Of the 12 participants from the first sampling group, two (16.6%) were male and 10 (83.3%) were female. Eight (66.6%) worked as clinical nurses, two (16.6%) as coordinating nurses, and another two (16.6%) as resident nurses. The mean age of the group was 37 years old. Regarding the schooling level, two (16.6%) were MSc and 10 (83.3%) were specialists. The mean time working in the service was three years.

Of the five participants from the second sampling group, two (40%) were male and three (60%) were female. The mean age was 41 years old. Regarding the schooling level, two (40%) were MSc and Ph.D. All five participants were graduates in Medicine and had some specialization. The mean time working in the service was three years. All of them held positions in the higher SMS management instances.

### Characterizing the Practical Approach to Care Kit as a technological innovation for nurses’ clinical performance in HIV management

The PACK emerged as a technology that allows reorienting nurses’ clinical practice in the management of HIV infection. It includes aspects related to work organization, duties and multi and inter-professional exchanges; clinical management by nurses; relationships with users and care efficacy; professional training and qualification; as well as aspects related to PHC as an organizer of the HIV care line.

Using the PACK during the appointments with people living with HIV allowed organizing the actions by means of flowcharts, targeting care to the person living with HIV, as indicated in the statement below:

*The PACK has a tab just for the care of HIV patients. So [...] we do the entire flowchart. Here in the diagnostic part, when the patient arrives, the test is performed. It was positive, what do you do? It was negative, what do you do? We have all the guidance, patient management part here* (N01)*.*

The technology also contributed to delimit the responsibilities and duties of professionals, physicians and nurses; for knowledge exchanges and information sharing among the team professionals, easing the work process and shared care.

*There is the part [...] when it’s emergency care, which professional is responsible for that, if it’s a doctor, if it’s a nurse* (N01)*.*

*So, this [the PACK] eased our work process, not only the nursing work, but the work of the team as a whole, for being able to share this care* (N04)*.*

The PACK guidelines assist in decision-making in relation to the prescriptions of medications and exams by nurses, in addition to guiding the organization of processes and dynamizing the practices during management of people with HIV. The tool allows the professionals to exercise their clinical practice based on evidence, providing safety in clinical management.

*[The PACK signals] which medications the nurse can prescribe, which exams, what the nurse’s and doctor’s duties are for each situation […]* (N01)*.*

*Yes, sometimes the patient arrives complaining [...] women, for example, “I’m taking this antiretroviral, can I take this contraceptive?” I go search in the PACK, and there I find this answer. It’s well structured, well organized* (N02)*.*

*Oh! The PACK is wonderful, I use it all the time! It helps a lot and dynamizes management, you go there [in the PACK] and everything is already done. And everything is based on evidence. So it gives you confidence in your clinical management* (N01)*.*

*The PACK experience itself shows the importance of having qualification and clinical management tools that guarantee patient safety, and from there you can train the nurse for clinical management* (M03)*.*

With the implementation of this technology it was possible to observe nurses’ empowerment in the exercise of their clinical practice, which, prior to the PACK, had their scope of action reduced in the monitoring of HIV infection. The PACK’s clinical guidelines for nurses encompass from diagnosis, evaluation of symptoms and the patient’s health condition, counseling, treatment adherence assessment, monitoring of adverse effects, aspects related to mental and sexual health and family planning, to prescription of tests, medications and immunobiologicals.

Nursing care proved to be more resolute after implementing the PACK, making health interventions more timely and granting greater autonomy to the professionals in the management of HIV infection. The professionals stated such advances through feelings of satisfaction:

*We follow-up with the doctor and have this empowerment in relation to the PACK, to really act in the treatment, to do this listening, welcoming, doing all the care, requesting some tests and monitoring the care with medications and side effects. I believe it’s a great experience!* (N05)*.*

*The PACK brings resoluteness to the follow-up offered by nurses [...]. For example, if the patient arrived at a time when it was suitable to request an exam, I couldn’t do that as a nurse [...], this limited my practice [...]. Since the PACK was implemented, today if the patient comes to the unit for HIV monitoring, he can consult with the nurse* (N04)*.*

*[The PACK] is for us not to make any decision and say: “Ah! It was me who made it.” No! If I made that decision, the professional at the other health center will make the same decision because we use this protocol* (N02)*.*

In addition, it was evidenced that using the PACK contributed to care resoluteness and allowed an approximation between the nurses and the people living with HIV.

*I’ve been working with this population since I’ve been in care, but I confess that there was a closer approach to the monitoring of people living with HIV from the moment we had the protocols and the PACK in place in the municipality [...]* (N04)*.*

*As we didn’t have this resoluteness [before the PACK], the doctor was the one taking care of these patients, [...] and the patients ended up looking for the doctor a lot more too. Now we can provide much more shared care in this sense, both in exams, medication and monitoring as a whole* (N04)*.*

The PACK also constituted a tool for permanent education in health, which provides a collective construction of diverse knowledge on the most frequent diseases in PHC, qualifying the care provided by the nurses.

*It’s supported by the best evidence [...]. I think that, in addition to offering this protocol based on the best evidence, the PACK also enables permanent in-service education* (N10)*.*

It was verified that, in addition to strengthening shared care among the team’s professionals, the PACK contributed to implementing the line of care for people with HIV, with emphasis on expanding access to PHC and regulation of the specialties.

*As a training tool, the PACK [...] helps to better address the line of care, create access, regulate access to the specialties [...]* (M03)*.*

**Figure 1:**
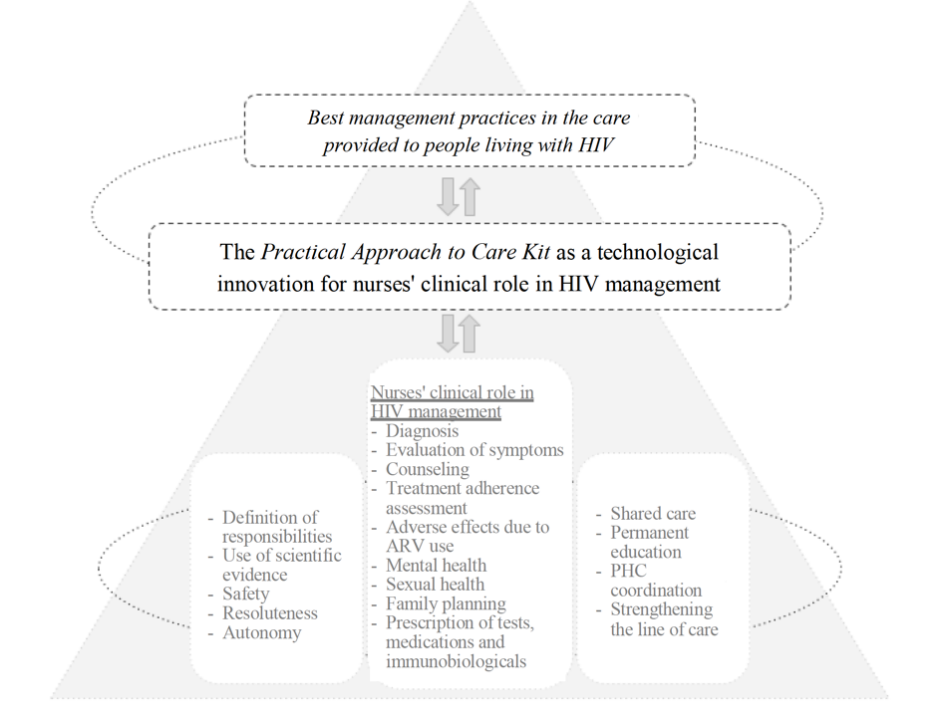
Diagram representing the elements that support the *Practical Approach to Care Kit* as a technological innovation for nurses’ clinical role in the management of the HIV infection in Primary Health Care

In synthesis, the core elements of the phenomenon under study signal use of the PACK as a technological innovation in management of the HIV infection. Figure 1 presents a synthesis of the main elements of the phenomenon under study. As can be seen, the data found made it possible to identify processes and practices indicating a reorganization of the nurses’ work process, with consequent advances for their clinical practice within the PHC scope.

## Discussion

The findings of this study allow characterizing the PACK as a non-material technological innovation of an incremental and process type^18^, which led to reorganization of the work performed in PHC services. Using the technology in HIV infection management enabled greater empowerment of nurses for the clinical follow-up of this health problem and favored interprofessional practices through shared care and decision-making.

Another study identified that PHC nurses from Florianópolis prefer to use Nursing protocols as a basis for carrying out their clinical practices, as they understand that they are frequently updated and guarantee greater legal support for their work^19^. The PACK’s clinical guidelines are conceptually aligned and updated with the municipal protocols, therefore, they constitute important technologies that guide the care of this health condition.

The clinical guidelines contained in the PACK evidence the specific activities of physicians and nurses alike, as members of the multiprofessional health team, guiding shared care and approaching interprofessional practices. In this logic, the literature indicates that nurses’ empowerment in the clinical practice is a core attribute for the development of competencies that involve decision-making in health, supported by the provisions of manuals, lines of care or specific or multiprofessional clinical protocols^20^ ^-^ ^21^. Clinical protocols and guidelines are crucial to provide safe and quality health care^22^.

The Brazilian government’s ministerial guidelines instruct decentralization of the care provided to people living with HIV to the PHC services, with institutional and matrix support from specialists in infectious diseases. This new care model aims at expanding the care offer, as well as at structuring care practices based on quality of life promotion, intervention in risk factors, and incorporation of programmatic and intersectoral actions. The new model includes reorganization of the care line and creation of a bond between professionals and people living with HIV, conferring greater resoluteness to PHC services^4^.

Such improvements demand qualification of the care provided by nurses; in this sense, using care guides and protocols in PHC, such as the PACK, expands nurses’ clinical performance and, consequently, the proportion of users who can be monitored by these professionals. A study carried out on PACK implementation records that its use provides more resolute care and resource optimization^23^. Another study, which analyzed implementation of the PACK in Brazil, pointed out that some nurses were resistant to expanding the scope of their practices and to the increase in workloads, as well as the physicians felt threatened with the expansion of nurses’ clinical performance. However, the explanation about delineation of the clinical roles and functions provided confidence and general acceptance toward the tool^10^.

The PACK was developed for the South African reality, where there were no physicians available to manage the most frequent diseases in PHC, including clinical management of HIV, which led to the expansion of nurses’ clinical role. International experiences of PACK implementation highlight good results achieved by using the technology^23^ ^-^ ^25^. In South Africa, improvements in care quality and in indicators such as prescription, referrals and case detection stand out, mainly in relation to communicable diseases. There are also good results related to health follow-ups and economic benefits, such as a reduction in the number and length of hospitalizations^23^.

In the same sense, in Pakistan, a study interviewed physicians, nurses and paramedics, and the results indicate high levels of acceptance of the PACK among health professionals, with emphasis on training initiatives and the protocol structure^25^. In Nigeria, the health professionals’ experience with the PACK was also positive in terms of ease of use, usefulness during the appointments, better ability to diagnose and manage patients, better cost-effectiveness (less polypharmacy and fewer exam requests), more confidence and appreciation of their professional performance^24^. Based on this diverse evidence, the PACK can be characterized as a successful innovation that expanded nurses’ clinical practice in the countries where it was implemented.

With regard to the PACK implementation process in Brazil, a study historicizes this process, noting that it began in 2014 and comprised different stages^10^. The first was the local engagement stage, which lasted approximately one year, where conversations were started to define the scope, financing, contracting and definition of those responsible for conducting the project in Brazil. At a second moment, the PACK was introduced, where the analysis of its principles and mentoring model was advanced, as well as a visit to South Africa to learn about the experiences using the tool^10^.

Subsequently, the translation stage was initiated with an adaptation to the context and public policies in Brazil and Florianópolis. From the beginning of this process to the complete graphic finalization of the material, 18 months of collaborative work took place, with participation of the translators, the Florianópolis SMS team and health professionals, who were able to contribute with their feedback on the tool. Subsequently, the PACK pilot implementation stage was initiated in 24 CSs for six months, when the professionals underwent training sessions and were able to use the PACK during their health care practices. Finally, the last implementation stage consisted in monitoring and evaluating the tool in the pilot CSs and in implementing in the other 25 CSs of the municipality, with an expected execution period of between one and five years^10^.

It is also worth noting that an earlier version of the PACK, known as *Practical Approach to Lung Health South Africa* (PALSA), was implemented in Minas Gerais/Brazil, Mexico, Malawi, Gambia and Botswana. However, the Florianópolis version was the first in which a mentoring model was used, featuring an improvement over previous versions, as mentoring was essential for adaptation and implementation of the technology^10^.

The PACK use analysis showed the importance and usefulness of HIV infection clinical management carried out in a shared way between physicians and nurses in PHC. The findings showed that the new clinical approach contributes to improving care for people living with HIV, positively influencing adherence to ARV treatment. In addition, the possibility of prescribing medications and requesting routine exams by nurses contributes to expanding the scope of the care provided in PHC. Development of the nurses’ clinical performance in PHC, with prescription of medications and exams, contributes to transforming care in the health teams’ context, maximizing care access and resoluteness^26^ ^-^ ^28^.

The results of the current study show that organizing the PACK in a flowchart format, with guidelines based on diverse scientific evidence, allowed for safe clinical decision-making during the care provided to people living with HIV. The scientific literature points out that using evidence-based instruments for care planning contributes to the adoption of safe and good quality practices, which is a priority recommendation for patient safety in health institutions^29^. The literature also records the importance of developing and incorporating low-cost technologies in PHC, to expand access, reduce costs and promote effectiveness of the services^30^.

A technological innovation can be understood as a change that breaks with traditional patterns in the production process in any sector of the economy^18^. The health field has been strongly influenced by the innovation process, including a wide diversity of material technologies and also new care modalities with a view to implementing improvements in health work processes and/or products^6^ ^,^ ^31^. The findings of this study allow conceptualizing the PACK as an innovative, non-material, process and incremental technology. This understanding is justified because it allowed realigning nurses’ clinical practice in HIV infection management, expressed by its contribution in organizing and targeting the care practices and in strengthening professional autonomy and permanent education, as well as contributing to qualifying the clinical practice in management of the HIV infection in PHC.

This research has limitations related to the virtual data collection method, as it did not allow observational analysis of the context and daily use of the PACK by the health professionals. Another limitation was the fact that no interviews were conducted with medical professionals, who also use the PACK in their daily work at PHC and share care with nurses, and therefore could contribute to analyzing the phenomenon.

The results and discussions pointed out in this study contribute to the scientific literature on nurses’ clinical role in management of the HIV infection, as well as they portray the advances experienced in PHC in Florianópolis with implementation of the PACK and decentralization of HIV clinical management, which still is recent throughout Brazil. This scenario can foster the development of policies and technologies to qualify care and support nurses’ clinical decision-making in PHC, expanding their clinical management skills and capabilities.

## Conclusion

It is concluded that the PACK is an important technological innovation used in nurses’ clinical practice for HIV management in PHC in Florianópolis. Its implementation restructured the nurses’ work process, preparing them to provide good quality care based on diverse scientific evidence, culminating in the expansion of these professionals’ clinical performance in the monitoring of HIV infection.

In this logic, it is suggested to expand use of the PACK to other Brazilian regions and municipalities, in order to strengthen the PHC services and contribute to structuring a decentralized care network for people living with HIV. In addition, the results highlight the importance of investing in the production of innovative technologies that strengthen the right to health and the provision of good quality care.
